# Poisons and Their Antidotes

**Published:** 1848

**Authors:** 


### ARTICLE VIII.

*Poisons and their antidotes.* From the American Medical Almanac.

Arsenic. *Arsenious Acid. Fowler's Solution. King's Yellow. Sheel's Green.*

*Symptoms.*—Metalic, austere taste in the mouth, violent burning pains in the region of the stomach, constriction of the pharynx and œsophagus, violent retching and vomiting and tenderness of the epigastrium, the matter vomited greenish or yellowish, often streaked with blood, severe gripings, purgings, and tenesmus, the stools being deep green or black, and horribly offensive ; sometimes excoriations of the anus ; the urine scant, red, and often bloody ; the pulse small, frequent and often intermitting ; difficult respirations and cold sweats ; countenance collapsed, eys red and sparkling, delirium, death.

*Treatment.*—If vomiting does not already exist an emetic of sulph. zinc may be exhibited and its effect, promoted by mucilaginous drinks. If sulph. zinc is not at hand, an emetic of mustard may be given. The best antidote is hydrated peroxide of iron, in doses of a table spoonfull or more every five minutes ; if the patient cannot swallow, it should be introduced by means of a stomach pump : if this antidote be not at hand, let the common red oxide mixed with water be used as a substitute. If the poison have been taken in the form of Fowler's Solution, copious draughts of lime water may be given. Counter irritants and opium may be used to relieve the pain and spasm. Bloodletting should *not* be employed, unless to allay the subsequent inflammation, or after the stomach has been throroughly evacuated.

Acids. *Sulphuric Acid.*

*Symptoms.*—Sour, styptic taste in the mouth, burning pain in the throat, gullet and stomach, increased by swallowing, nausea, vomiting, and horrible fœtor of the breath; matter vomited tinged both by arterial and venous blood, and effervesces if mixed with carbonate of lime; difficult respiration, croupy cough, small, contracted, and feeble pulse.

*Treatment.*—Dilute largely with milk mixed with the carbonate of potassa, lime, or magnesia. In the absence of these, with soap suds, infusions of wood ashes, white of eggs, milk, or oil. Water should not be allowed, in consequence of the heat generated upon its mixture with sulphuric acids.

Nitric Acid. *Aqua Fortis.*

*Symptoms.*—Much the same as those of sulphuric acid. If the acid be strong and the dose large, almost immediate death follows; if it be weak, the patient may linger for a considerable time, vomiting at intervals, shreds of membranes which have an almost insupporting fœtor; obstinate constipation; yellow spots upon the skin where the acid has fallen.

*Treatment.*—Carbonate of magnesia or lime, in water, or any bland fluid; then evacuate the stomach by large draughts of demulcent fluid and treat the secondary symptoms on general principles.

Oxalic Acid. *Salt of Lemons.*

*Symptoms.*—Burning pain in the stomach, nausea, and severe but ineffectual efforts to vomit, great dilatation of pupils, vertigo, convulsions, death.

*Treatment.*—Give large quantities of chalk, whiting, or magnesia or its carbonate, made into a cream with water and freely exhibited. In the absence of these antidotes, administer copious draughts of warm water, at the same time promoting vomiting by tickling the throat. Avoid the alkalies, potash and soda or their carbonates, since the salts which they form with oxalic acid are as poisonous as the acid itself.

Muriatic Acid. *Hydrochloric Acid.*

*Symptoms.*—Same as sulphuric acid. It is said by Orfila, that when muriatic acid is the poison taken, thick white fume of a sharpe penetrating odor, similar to that exhaled by the acid issues from the mouth.

*Treatment.*—Same as sulphuric acid.

Mercurials. *Corrosive Sublimate.*

*Symptoms.*—Acrid, metallic, astrigent taste, sensation of fulnesss and burning in the throat, burning pain in the stomach and intestines; nausea, vomiting and purging, often of bloody matter; pulse small, quick and hard; frequent faintings, great prostration, sometimes coma, convulsions and death.

*Treatment.*—Large quantities of albumen in some form or other, as white of egg, for instance, must be freely administered; if this cannot be had, wheat flour beaten up with soap-suds may be used. Bleeding is requisite if the pulse be quick and hard; inflammatory symptoms to be treated on general principles.

White Mercury. *White Precipitate.*

*Symptoms and Treatment* same as corrosive sublimate.

Turbith Mineral. *Sulphate Binoxide of Mercury.*

*Symptoms and Treatment* same as corrosive sublimate.

Other Mineral Irritants. *Tartar Emetic.*

*Symptoms.*—Nausea and severe vomiting, hiccough, burning pain in the pit of the stomach, griping and purging, sense of tightness in the throat, small, frequent, and hard pulse, difficult respiration, vertigo, great prostration, insensibility, death.

*Treatment.*—If vomiting have not occurred, it should be produced by copious draughts of warm water, and tickling the faucus with a feather. Dilute freely with a tepid infusion of galls, Peruvian bark, oak bark, or green tea, to form an insoluble tannate. Powdered yellow bark may be used until the infusion is prepared. Opium is highly useful in allaying the pain and excessive evacuation.

Sulphate of Iron. *Green Vitriol. Copperas.*

*Symptoms.*—Griping pains in the stomach and abdomen constant vomiting and purging, violent pain in the throat, coldness of skin and feebleness of pulse.

*Treatment.*—Carbonate of soda or magnesia given freely is the best antidote. Evacuating the stomach by means of emetics of sulph. of zinc, and inflammatory symptoms treated on general principles.

Chloride of Tin. *Spirits of Tin. Dyer's Spirits.*

*Symptoms.*—The same as those from other irritant poisons, and a peuliar tanned appearance of the villous coat of the stomach.

*Treatment.*—Milk acts as an antidote to this poison and should be drunk copiously. Vomiting should then be excited.

Sub-acetate of Lfad. *Sugar of Lead.*

*Symptoms.*—A burning, pricking sensation in the throat, with dryness and thirst, irritation of the alimentary canal, spasms, vomiting, and often colic; rigidity of the abdominal muscles, cramp, obstinate constipation, urine diminished, saliva increased. When the case is protracted, paralysis of the upper extremities.

*Treatment.*—This consists in the free exhibition of solution ot the alkaline sulphates, either soda or magnesia. Phosphate of soda is also an antidote. Carbonates should be avoided, as carbonate of lead is poisonous. If vomiting do not exist, emetics of sulph. of zinc should be given. In the chronic form, or colica pictonum, purgatives, and anodynes are resorted to.

CARBONATE OF POTASH. *Pearl ash. Salt of Tartar.*

*Symptoms.*—Those common to other irritant poisons. The matter vomited effervesces with acids.

*Treatment.*—The patient should be made to swallow from time to time, draughts of vinegar and water, lemon juice, or other vegetable acids. The fixed oils are also good antidotes, such as the castor, linseed, olive, and almond; they form a soap by uniting with the alkali and thus destroy its caustic effects.

NITRATE OF SILVER. *Lunar Caustic.*

*Symptoms.*—Nearly the same as those produced by corrosive sublimate; the pain and burning in the stomach, however, are more severe.

*Treatment.*—A strong solution of chloride of sodium or common salt, is the best antidote; it forms an innocuous chloride of silver; then evacuate the stomach by an emetic, and treat the inflammatory symptoms on general principles.

VERDIGRIS. *Sub-acetate of Copper.*

*Symptoms.*—Very similar to those produced by arsenic; vomiting of a green colored liquid, and diarrhœa are the most prominent symptoms; coppery eructations, and taste in the mouth.

*Treatment.*—The efforts to vomit should be promoted by the free exhibition of warm warter, milk, or any mucilaginous drink; the chemical antidote for the preparation of the copper is albumen, if not at hand, wheaten flour and water, or milk. Vinegar should not be given; iron filings mixed with water may be given with good effect; very strong coffee with plenty of sugar, also acts as an antidote by decomposing the salt of copper.

SULPHATE OF COPPER. *Blue Vitriol.*

*Symptoms.*—Violent vomiting of matter remarkable for being of a blue or green color, sometimes containing broken crystals of the blue vitrol; pain in the abdomen and diarrhœa.

*Treatment.*—Same as for sub-acetate of copper.

SULPHATE OF ZINC. *White Vitriol.*

*Symptoms.*—Violent vomiting, astringent taste in the mouth, a sensation of choaking, burning pain in the stomach and lower belly, quickened pulse, paleness and shrinking of the features and coldness of the extremities. Death rarely follows, owing to the prompt emetic action of the poison.

*Treatment.*—Assist the vomiting by copious draughts of warm water; carbonate of soda in solution, to decompose the sulph. of zinc. Let the patient drink freely of milk, or albumen, which partially decomposes the poison, and renders it more inert.

NITRATE OF POTASSA. *Nitre. Saltpetre.* Sometimes taken in mistake for Glaubers' salts.

*Symptoms.*—Nausea, vomiting and excessive purging, accompanied by bloody stools, and excruciating pain in the bowels; sensation of intense heat in the stomach, dyspnœa, cold extremities, syncope, convulsions, death.

*Treatment.*—Empty the stomach as rapidly as possible, either by emetics or the stomach pump; then let the patient drink freely of milk, flaxseed tea, or other bland mucilaginous drinks.

NITRATE OF BISMUTH. *Pearl Powder.*

*Symptoms.*—Similar to those produced by corrosive sublimate, general inflammation of the whole alimentary canal, vomiting, sensation of great heat in the chest, and difficulty of breathing.

*Treatment.*—Large draughts of milk, which is coagulated by the poison thus entangling it and enabling it to be expelled from the stomach; to be followed by emetics. Inflammation to be treated on general principles.

PHOSPHORUS.

*Symptoms.*—Violent pain in the stomach, with a hot garlicky taste in the mouth, vomiting, diarrhœa, great excitement of the arterial and nervous system, convulsions, and death.

*Treatment.*—Large draughts of water, or any mucilaginous fluid, so as to envelope the phosporous and impede the combustion. An emetic to be properly administered; magnesia may be mixed with the fluid to neutralize the phosphorus and and phosphoric acids which may be formed. Oily and fatty substances should be avoided.

VEGETABLE IRRITANTS. HELLEBORE. CONIUM OF HEMLOCK. BELLADONNA OR NIGHTSHADE. DATURA STRAMONIUM OR THORN APPLE. ACONITE OR MONKSHOOD.

*Symptoms.*—The symptoms of these poisons resemble each other so much, that they may be classified under the same head; stupor, numbness, heaviness in the head, nausea, sometimes vomiting, at others the stomach and bowels are so paralyzed, that vomiting can scarcely be produced by the most powerful emetics; a sort of intoxication, pupils dilated, sometimes delirium, redness and tumefaction of the face, convulsions and death. The same results follow when these substances are applied to wounds.

*Treatment.*—Empty the stomach as rapidly as possible, by emetics of tartarized antimony, sulphate of zinc or sulphate of copper. Evacuate the bowels by active purgatives and injections, and *follow* these by large doses of vinegar and water and other vegetable acids. After the vomiting, strong coffee proves very efficacious in removing the insensibility. If coma or apoplexy be present, after the evacuation of the stomach, treat it by blood-letting, and revellents.

SAVINE. *Oil of Savine.*

*Symptoms.*—All those of high excitement, with very acute pain in the stomach and bowels ; nausea, vomiting, excessive purging and convulsion, and abortion in pregnant women.

*Treatment.*—Evacuate the stomach by copious dilutions with mucilaginous fluids, and keep down inflammatory symptoms by the lancet and antiphlogistics.

ERGOT. *Secale Cornutum. Spurred Rye.*

*Symptoms.*—In large dozes, nausea and vomiting, dryness of the throat, great thirst, uneasiness or actual pain in the abdomen, occasionally alvine evacuations, weight and pain in the head, giddiness, stupor and dilatation of the pupils, diminished frequency and fulness of the pulse, paleness and lividity of the face, and when its use is long continued, gangrene. It is frequently given in poisonous doses to produce abortion.

*Treatment.*—First evacuate the poisons from the alimentary canal, by the use of emetics or purgatives. Chloride water has been recommended to decompose the ergotin, or active principle ; in the absence of this, nitro-hydrochloric acid properly diluted might be exhibited. Subsequent treatment on general principles.

CASTOR OIL SEED.

*Symptoms.*—Vomiting, purging, and griping, of great violence, burning pain in the stomach and bowels, tenderness on pressure, hiccough, faintness, and small, feeble pulse.

*Treatment.*—Evacuate the stomach by an emetic, and treat the inflammatory symptoms by diluents, anodynes, and antiphlogistics generally.

OPIUM, *and its preparations. Laudanum. Paregoric. Godfrey's Cordial. Dalby's Carminative. McMunn's Elixir. Morphia and its salts.*

*Symptoms.*—Drowsiness and stupor, followed by delirium, pallid countenance, sighing, deep and stertorous breathing, cold sweats, coma and death.

*Treatment.*—The stomach pump should be used as soon as possible when the liquid poisons or powdered opium have been taken ; when not at hand, give from one to two scruples sulph. of zinc, or five to fifteen grs. sulph. copper ; if these cannot be readily had, a teaspoonful of powdered mustard, or a tablespoonful of common salt, dissolved in a tumbler of water. Emesis should be promoted by tickling the fauces with a feather. In using the stomach pump, it is better to inject astringent infusions, as the infusions of galls, which has the effect of neutralizing the morphia. After the stomach has been evacuated, the cold dash is an effective means of rousing the patient; the subsequent narcotic effect may be obviated, by the administration of the vegetable acids, hot strong coffee, brandy, or ammonia. The patient should also be kept in motion and on no account allowed to sleep. Cupping to the temples is sometimes useful, as well as the warm bath where the drowsiness is very great. As a last resort, artificial respiration is sometimes useful in averting the fatal termination.

CAMPHOR.

*Symptoms.*—Great excitement of the brain and nervous system, vertego, great anxiety, insensibility, vomiting, small pulse, difficult respiration, cold sweat, and convulsions.

*Treatment.*—If solid camphor has been used, an emetic should be administered, and wine and opium exhibited at short intervals until the symptoms abate.

HYDROCYANIC ACID. *Prussic acid. Essential oil of almonds. Laurel water.*

*Symptoms.*—Nausea, giddiness, debility, vertigo, and loss of sight, weight and pain in the head, eructations having the flavor of the acid, dyspnœa, small vibrating pulse, spasms, dilated pupil, convulsions and death.,

*Treatment.*—There are four principal agents to be relied on in the treatment of poisoning by this acid, viz: *Chlorine, ammonia, cold affusion, and artificial respiration. Chlorine* water may be given in doses of f. ʒ ij. in f. ℥ j. of water, or 30 or 40 drops of the solution of chloride of soda in a little water; the patient should at the same time inhale air impregnated with chlorine gas. *Ammonia* is also an antidote, but it should not be employed in a very concentrated form; it may be administered either by inhalation or in substance. *Cold affusion* being always at hand, may be applied immediately; a stream of cold water should be poured from a height upon the head and spine for some minutes; cold water may also be dashed upon the head and face. *Artificial respiration* should never be omitted. This can be easily effected, by making powerful pressure with both hands on the anterior surface of the chest, the diaphragm being at the same time pushed upward by an assistant, inspiration being effected by the mere removal of the pressure and the consequent resiliency of the ribs.

CARBONIC ACID GAS. *Fumes of burning charcoal.*

*Symptoms.*—Same as those of apoplexy, or narcotic poisoning; great drowsiness, difficulty of respiration, and suffocation; features swelled and face bluish, as in cases of strangulation.

*Treatment.*—Remove the patient at once into the open air, elevate the head, and pour cold water upon it, bleed either by cups to the head, or by opening a vein, apply friction to the thorax, and revellents to the feet. If these means fail, then make use of artificial respiration. Administer stimulants cautiously, as soon as the patient can swallow.

ALCOHOL. *Brandy, Wine, Spirits, &c.*

*Symptoms.*—Those of narcotic poisons; insensibility with apoplexy or paralysis. Countenance swollen and of a dark red color, and stertorous respiration. The poison can often be detected by the smell.

*Treatment.*—If the patient can swallow, administer an emetic of sulphate of zinc or tartar emetic; if not, evacuate the stomach, by means of the stomach pump and apply cold affusions to the head, which should be elevated; if these means fail, blood-letting should be resorted to, either by cups, or by opening a vein, and generally, the jugular, should be selected. Artificial respiration is necessary in some instances.

